# Exploring the Chemical Composition of Female Zucchini Flowers for Their Possible Use as Nutraceutical Ingredient

**DOI:** 10.3390/antiox12122108

**Published:** 2023-12-13

**Authors:** Luigi Castaldo, Sonia Lombardi, Luana Izzo, Alberto Ritieni

**Affiliations:** Food Laboratory, Department of Pharmacy, University of Naples Federico II, Via Domenico Montesano 49, 80131 Naples, Italy; luigi.castaldo2@unina.it (L.C.); sonia.lombardi@unina.it (S.L.); alberto.ritieni@unina.it (A.R.)

**Keywords:** polyphenols, bioactive compounds, *Cucurbita pepo* L., nutraceuticals, bioaccessibility

## Abstract

The zucchini (*Cucurbita pepo* L.) plant is well known for its fruits; however, its edible flowers appear to contain several active molecules, including polyphenols, which display poor bioaccessibility after gastrointestinal digestion (GiD). This study explores the bioaccessibility of polyphenols and antioxidant capacity within zucchini flower extracts during simulated GiD. Two nutraceutical formulations, non-acid-resistant (NAcR) and acid-resistant (AcR) capsules containing an aqueous extract of zucchini flowers, were employed in this investigation. Additionally, high-resolution mass spectrometry (Q-Orbitrap HRMS) was utilized for a comprehensive analysis of their polyphenolic constituents. Predominantly, rutin and isorhamnetin-3-rutinoside were the most prevalent compounds detected in the samples (514.62 and 318.59 mg/kg, respectively). Following in vitro GiD, the extract encapsulated in AcR capsules exhibited enhanced bioaccessibility during both the duodenal (189.2 and 162.5 mg GAE/100 g, respectively) and colonic stages (477.4 and 344.7 mg GAE/100 g, respectively) when compared with the extract encapsulated in NAcR capsules. This suggests that gastric acidity adversely impacted the release of polyphenols from NAcR capsules. In conclusion, the aqueous zucchini flower extract emerges as a promising and readily accessible source of dietary polyphenols. Moreover, the utilization of AcR capsules presents a potential nutraceutical formulation strategy to improve polyphenol bioaccessibility, enhancing its applicability in promoting health and well-being.

## 1. Introduction

Edible flowers have gained increasing popularity as consumers have sought innovative natural sources of bioactive compounds and the world has discovered the possible potential of flowers, although historically they have mostly been used for their smell and visual appeal [1]. This is due to their distinctive sensory properties, characterized by their vivid coloration, pleasant aroma, and flavor [2]. Several scientific studies have suggested that edible flowers contain appreciable levels of antioxidants that could be used effectively in the food industry [3]. Antioxidant consumption has been linked to a wide range of health-promoting effects, possibly preventing several age-related diseases including type 2 diabetes, cancer, and heart disease [4]. Furthermore, they play a crucial role in maintaining and promoting a healthy and productive life [5]. Plants, herbs, fruits, and vegetables represent the main sources of dietary antioxidants [6].

In addition, the use of antioxidant compounds recovered from agro-industrial material has increased during the last decade [7,8]. In fact, plant materials contain appreciable amounts of antioxidant molecules that may be used effectively as innovative ingredients in the formulation of dietary supplements, functional foods, natural colorants, and nutraceutical products [9]. In recent years, there has been a growing interest in developing sustainable methods for the extraction of bioactive compounds from agro-industrial materials [10,11]. Among these, the use of simple aqueous extraction is considered to be the most environmentally friendly approach, due to its low cost, simplicity, and minimal use of organic solvents [12,13].

The *Cucurbita pepo* L. plant, typically called zucchini, is well known for its edible fruits, which are grown extensively all over the world [14]. However, recent research has revealed that the flowers of this plant also contain bioactive molecules, specifically polyphenols, which may have positive health effects [15]. Polyphenols are a heterogeneous group of compounds reported in many plant-based foods and are well known for their important antioxidant properties [16]. These active molecules play a pivotal role in protecting cells from free radical damage that could be caused by free radical species [17,18]. Several scientific studies have reported that it is crucial for polyphenols to reach the target tissue to effectively exert their positive effects on human health [19]; the fundamental factor for maximizing the bioactive properties of polyphenols is represented by their bioaccessibility [20]. Nevertheless, the absorption of polyphenols is strongly influenced by both human digestive enzymes and the intestinal microflora [21]. The gastrointestinal digestion (GiD) process is complex and involves numerous factors that can affect the bioaccessibility of polyphenols, including changes in pH and temperature, bacterial microflora, and the activity of digestive enzymes [22]. These factors can also modify the potential health benefits associated with polyphenol intake [23]. The recent literature has suggested potential strategies to overcome this limitation [24]. The use of nutraceutical forms to enhance the bioaccessibility of polyphenols is considered a reliable method to preserve the chemical characteristics of active compounds, which could be altered after the GiD process [25].

On the other hand, the polyphenolic components of zucchini flowers have received limited investigation regarding their potential as an innovative ingredient in nutraceutical and dietary supplement formulations. Recently, the ethanolic extracts of zucchini flowers showed high concentrations of flavonoids, including rutin and quercetin glycoside, detected by high-pressure liquid chromatography (HPLC) analysis [26]. In addition, the work of Dujmović et al. [27] highlighted an appreciable amount of flavonoids found in zucchini flowers obtained with ethanol extraction, reaching 73.6 mg of total flavonoids per 100 g of fresh sample.

Regarding analytical methods, with the introduction of ultra-HPLC, the LC approach has evolved with significant developments [28]. For the detection and quantification of natural compounds, particularly polyphenols present in herbal-based products, the integration of UHPLC with high-resolution mass spectrometers (HRMSs), such as the Q-Orbitrap, has proven to be an effective approach [29,30]. This cutting-edge instrument displays high specificity and sensitivity, enabling precise quantification through accurate mass measurement [31].

Therefore, the aim of the present study was to perform an in-depth investigation of the polyphenolic profile of the aqueous extract of female zucchini flowers using UHPLC Q-Orbitrap HRMS analysis. The antioxidant activity and bioaccessibility of polyphenols during the in vitro GiD process were also evaluated, employing two nutraceutical formulations, composed of non-acid-resistant (NAcR) and acid-resistant (AcR) capsules, which contained the water extract of zucchini flowers. The objective of this evaluation was to obtain valuable insights into the efficacy of this novel source of active compounds in two different nutraceutical products.

## 2. Materials and Methods

### 2.1. Chemicals and Reagents

Water, methanol, formic acid, and hydrochloric acid were purchased from Merck (Darmstadt, Germany). The standards of polyphenols (purity > 98%) were purchased from Sigma Aldrich (Milan, Italy), and included quinic acid, protocatechuic acid, chlorogenic acid, catechin, epicatechin, caffeic acid, p-coumaric acid, vitexin, apigenin-7-O-glucoside, ferulic acid, naringin, quercetin-3-galactoside, rutin, diosmin, kaempferol-3-glucoside, isorhamnetin-3-rutinoside, myricetin, daidzein, quercetin, naringenin, luteolin, kaempferol, genistein, and apigenin. Potassium chloride, monosodium phosphate, sodium carbonate, potassium thiocyanate, sodium sulfate, sodium chloride, potassium persulphate, sodium bicarbonate, 2,2-azinobis (3-ethylbenzothiazoline-6-sulphonic acid) diammonium salt, 1,1-Diphenyl-2-picrylhydrazyl, Folin–Ciocalteu reagent, and gastrointestinal digestion enzymes were purchased from Merck (Darmstadt, Germany). All chemicals and reagents were of analytical grade.

### 2.2. Sampling

*Cucurbita pepo* L. female flower packs were acquired from local supermarkets located in the Campania region, Southern Italy. Plants of zucchini were cultivated at multiple locations within the Lazio region, Central Italy. The flowers underwent a rapid cold-water wash and were subjected to lyophilization through freeze-drying. The resulting samples were pulverized into a fine powder using laboratory milling methods and then stored at −80 °C until analysis.

### 2.3. Polyphenolic Extraction

The protocol used for extracting polyphenolic compounds from female zucchini flowers was based on a previously employed procedure [32]. Freeze-dried samples weighing 5 g were suspended in 100 mL of deionized water, and the mixture was subjected to shaking in a water bath for 30 min at 80 °C, with stirring at 120× *g*. Then, the mixture was sonicated for 25 min in the dark at 4 °C, followed by centrifugation at 3800× *g* for 4 min. All the resulting supernatant was collected, and the pellet was re-extracted using the same procedure. The supernatants obtained from both extractions were combined, lyophilized, and ground into a fine powder suitable for capsule formulation. The lyophilized polyphenolic extract powder was precisely weighed, mixed thoroughly, and filled into AcR (pharmaceutical-grade hydroxypropyl methylcellulose) and NacR (pharmaceutical-grade gelatin capsules) capsules by hand under controlled conditions to prevent contamination. Subsequently, the capsules were sealed manually to ensure integrity and prevent leakage. The capsules contained 500 mg of zucchini flower polyphenolic extract. However, in the control capsules (CT), 0.500 g of cellulose was utilized in place of the polyphenolic extract of the zucchini flower.

### 2.4. Advanced Analytical Techniques: Combining UHPLC with Orbitrap HRMS for Accurate Compound Identification

The samples were appropriately diluted with 80% MeOH and filtered through 0.2 µm syringe filters before analysis. Chromatographic separation was conducted using a UHPLC instrument (Dionex UltiMate 3000). The column employed for chromatography separation was a Kinetex F5 column (Phenomenex, Torrance, CA, USA), which was thermostated at 25 °C and had dimensions of 50 × 2.1 mm and 1.7 µm particle size. The eluent phases were water (A) and methanol (B), both of which contained 0.1% formic acid. The injection volume was 5 µL. The separation was performed using a gradient elution method. The separation was initiated with 100% phase A for 1 min, followed by a 2 min gradient to 20% phase A. The gradient was then further decreased to 0% phase A in 3 min. After this, the gradient was shifted back to its initial position in 2 min, with 100% phase A. The time constant was set at 25 ms, and the acquisition rate was configured to 30 Hz. The dwell volume of the system was set at 0.1 mL.

To perform detection, a Full MS mode was used with a negative mode Q-Exactive Orbitrap mass spectrometer (Thermo Fisher Scientific, Waltham, MA, USA). The instrument was set up with the following parameters: Microscan 1, resolution power of 70,000 FWHM, sheath gas flow rate of 18, AGC target of 1 × 10^6^, auxiliary gas of 3, maximum injection time of 200 ms, scan range of 80–1000 *m/z*, S-lens RF level of 60, capillary temperature of 320 °C, auxiliary gas heater temperature of 350 °C, spray voltage of 3.5 KV. A mass tolerance of 5 ppm was applied for detection. Data processing was carried out with Xcalibur software 3.1.66.19.

### 2.5. Simulated Gastrointestinal Digestion

The aqueous extracts obtained from female zucchini flower and incorporated into AcR and NAcR capsules for nutraceutical use were analyzed in vitro to assess the effects of GiD on their antioxidant activity and polyphenol bioaccessibility. The digestive process was simulated using the method established by the INFOGEST network [33]. The simulated salivary, gastric, and intestinal fluids (SSF, SGF, and SIF) were prepared following the protocol previously described by Minekus et al. [33].

In brief, the employed INFOGEST protocol was as follows. To replicate the oral phase, the AcR and NAcR capsules containing the aqueous extract of female zucchini flowers were suspended in a solution consisting of 3.5 mL of SSF, 975 µL of deionized water, 0.5 mL of α-amylase solution, and 25 µL of 0.3 M CaCl_2_ (H_2_O)_2_. The mixture was then neutralized to a pH of 7.0 ± 0.2 using 1 M sodium hydroxide and subjected to incubation at 37 °C and 120× *g* for 2 min in a thermostated shaker bath.

To mimic the physiological gastric condition, a combination of 7.5 mL of SGF, 5 µL of 0.3 M CaCl_2_ (H_2_O)_2_, 695 µL of water, and 1.6 mL of pepsin solution were carefully homogenized. The resulting mixture was subjected to an incubation period of 120 min at 37 °C with agitation at 120× *g*. Subsequently, the pH of the solution was adjusted to 3 using 1 M hydrochloric acid.

To replicate the conditions of the intestinal phase, a combination of 5 mL pancreatin solution, 1300 µL of water, 40 µL of 0.3 M CaCl_2_ (H_2_O)_2_, 2.5 mL bile salt solution, and 11 mL of SIF were thoroughly mixed. The pH of the resulting solution was raised to 7. The sample was subjected to an incubation period of 2 h at 37 °C with agitation in a thermostated shaker bath at 120× *g*.

Finally, samples were subjected to a colon stage simulation procedure according to a previously reported protocol [34]. The procedure involved adding 5 mL of Pronase E solution (1 mg/mL) to the pellets and incubating the mixture at 37 °C and pH 8 for 60 min. Subsequently, 5 mL of water and 150 µL of Viscozyme L were added to the solution, and the mixture was incubated for 16 h at 37 °C and pH 4. After incubation, 500 µL of the resulting supernatant was collected, freeze-dried, and stored at −18 °C for further analysis.

### 2.6. Quantification of Antioxidant Capacity of the Nutraceutical Formulation Enriched with Female Zucchini Flower Extract Subjected to Gastrointestinal Digestion

The antioxidant activity of female zucchini flower extract in AcR and NAcR capsules exposed to GiD was determined through two different spectrophotometric assays, and the results were displayed as mmol of Trolox per kg of sample.

#### 2.6.1. ABTS Test

The ABTS assay was conducted following the protocol described by Re et al. [35]. In brief, 2.5 mL of ABTS (7 mM) was combined with 44 µL of potassium persulfate (2.5 mM), mixed thoroughly, and then kept at room temperature for 16 h. The resulting solution was subsequently diluted with ethanol until an absorbance value of 0.70 (±0.02) at 734 nm was obtained. A volume of 100 µL of appropriately diluted samples was added to 1 mL of the ABTS radical working solution, and the reduction in absorbance was recorded after 3 min at 734 nm.

#### 2.6.2. DPPH Test

The DPPH assay was performed following the protocol proposed by Xie et al. [36]. In brief, 1 mg of DPPH was diluted in MeOH until the absorbance value reached 0.90 (±0.02) at 517 nm. Then, 200 µL of the samples was mixed with 1 mL of the DPPH radical working solution. After incubating for 10 min, the reduction in absorbance was recorded at 10 min.

### 2.7. Total Phenolic Content

The total phenolic content (TPC) of the samples was determined using a modified version of the method described by Gao et al. [37]. Briefly, 125 µL of the samples was mixed with 500 µL of deionized water and 125 µL of 2 N Folin–Ciocâlteu reagent, followed by incubation for 6 min at room temperature. Subsequently, 1 mL of deionized water and 1.25 mL of a 7.5% Na_2_CO_3_ solution were added to the mixture and thoroughly mixed. Absorbance at 760 nm was recorded after 90 min. The results were expressed as milligrams of gallic acid equivalents (GAEs) per 100 g of sample.

### 2.8. Statistical Analysis

The data were analyzed using Stata 12 software (STATACorp LP, College Station, TX, USA). The differences among groups were assessed through Tukey’s test with a significance level of *p*-value ≤ 0.05. The results were presented as the mean ± standard deviation, and all experiments were conducted in triplicate.

## 3. Results

### 3.1. Identification of Polyphenolic Compounds by UHPLC Coupled with Orbitrap HRMS Analysis

The identification of polyphenolic compounds in female zucchini flower extract was carried out using UHPLC-Q-Orbitrap HRMS analysis. The present work investigated a total of 24 different analytes. Data for mass parameters such as adduct ion, measured and theoretical mass, accuracy, sensitivity, and retention time (RT) are displayed in Table 1. Experiments were carried out in negative ESI^−^ mode, and full-scan HRMS was used to monitor the results. The structural isomers epicatechin and catechin (*m/z* 289.07175), luteolin and kaempferol (*m/z* 285.04046), and genistein and apigenin (*m/z* 269.04554) were identified by comparing the RTs of the real standards with RTs of the peaks and data obtained from the literature [38]. Sensitivity was assessed through the limit of detection (LOD) and limit of quantification (LOQ). The LOD was determined as the minimum concentration at which the molecular ion could be identified with a mass error below 5 ppm. The LOQ was established as the lowest analyte concentration that generated a chromatographic peak with precision and accuracy below 20%.

### 3.2. Quantifying Polyphenolic Compounds in Aqueous Extract of Female Zucchini Flowers via UHPLC-Q-Orbitrap HRMS

A detailed analysis was performed to investigate the specific polyphenols in the extract obtained from zucchini flowers, employing the UHPLC-Q-Orbitrap HRMS approach. Calibration curves in triplicate at nine different concentration levels were employed to measure the investigated compounds. Each calibration curve was prepared in triplicate. These calibration curves displayed strong linearity in the quantification process, with regression coefficients consistently exceeding 0.990. The results of our quantitative analysis are presented in Table 2, showing the average content of each polyphenol in milligrams per kilogram (mg/kg) of the extract, along with their corresponding standard deviations. Quantitative analysis revealed that the aqueous extract obtained from zucchini flowers contains a sum of the investigated polyphenolic compounds of up to 1026.08 mg/kg of extract. Compared to other polyphenolic components, rutin was identified as the prevalent one, comprising approximately 50% of the total polyphenols found in the extracts. Furthermore, the main flavonoids, including isorhamnetin-3-rutinoside, quercetin-3-galactoside, kaempferol-3-glucoside, myricetin, quercetin, kaempferol, and apigenin were also quantified, as detailed in Table 2. Additionally, our investigation identified *p*-coumaric acid as the primary phenolic acid and other compounds present in the tested extract, as outlined in Table 2.

### 3.3. Evaluating Polyphenol Release in Nutraceutical Formulations with Aqueous Extract of Zucchini Flowers

An in vitro simulated GiD was conducted on the water-based extracts of zucchini flowers enclosed in AcR and NAcR formulations. This analysis aimed to clarify which formulation could effectively preserve and improve the bioaccessibility of polyphenols under simulated GiD, thus providing information on their suitability as delivery systems for these active compounds. Throughout each phase of the in vitro GiD process, we employed the Folin–Ciocâlteu method to investigate TPC levels. Notably, the TPC of the extract in AcR capsules exhibited a significant increase (*p*-value < 0.05) during the duodenal phase, measuring 189.2 mg GAE/100 g compared to 162.5 mg GAE/100 g for NAcR samples (Table 3). Similarly, in the colonic phase (comprising the Viscozyme L phase and Pronase E phase), the extract in AcR capsules displayed a significantly higher TPC value (*p*-value < 0.05) of 477.4 mg GAE/100 g compared to 344.7 mg GAE/100 g for NAcR samples. Regarding control samples, phenolic compounds were not detected in CT-AcR and CT-NAcR after all stages of the GiD process (Table S1, Supplementary Materials). Furthermore, non-encapsulated extracts were also exposed to GiD digestion; the results are shown in Table S2.

### 3.4. Evaluating Antioxidant Capacity of Zucchini Flower Aqueous Extracts in AcR and NAcR Capsules

We evaluated the antioxidant capacity of aqueous extracts of zucchini flowers that were encapsulated in both AcR and NAcR formulations. Two different assays, ABTS and DPPH, were employed at various stages of GiD to determine the most effective delivery method for preserving antioxidant compounds during this process. The results, expressed as mmol Trolox equivalents per kg of the sample at each GiD phase, are presented in Table 4.

Throughout all experiments, the antioxidant activity of both AcR and NAcR capsules exhibited a significant decline (*p*-value ≤ 0.05) compared to the non-digested samples across all GiD stages. Interestingly, during the colonic stage, encompassing combined data from the Viscozyme L phase and Pronase E phase, both AcR and NAcR capsules consistently demonstrated the highest recorded antioxidant capacity throughout the simulated GiD process. Table 4 clearly illustrates that AcR samples consistently exhibited the most robust antioxidant activity during this phase in all conducted spectrophotometric tests. Moreover, we thoroughly assessed the antioxidant capacity of the non-encapsulated extract throughout the simulated GiD, with results presented in Table S3. The TPC values obtained during GiD were further correlated with ABTS and DPPH data, as detailed in Table S4. Additionally, for the control samples, the data recorded for CT-AcR and CT-NAcR capsules during the simulated GD can be found in Table S5.

## 4. Discussion

This study aimed to investigate the active compounds, particularly polyphenols, within female zucchini flowers. The aim was to enhance the value of this agricultural industry material through a comprehensive examination. To achieve this, we employed a simple aqueous-based extraction method, ensuring the production of a food-grade extract from this novel natural resource.

In summary, our findings underscore the potential of zucchini flowers as a promising and innovative source of active compounds. Notably, we identified several phenolic compounds from the cinnamic and benzoic acid groups, including caffeic acid, chlorogenic acid, ferulic acid, *p*-coumaric acid, quinic acid, and protocatechuic acid. Additionally, we found a wide range of flavonoids in water-based extracts of zucchini flowers, including isoflavones, flavonols, flavanones, flavanols, and flavones, with rutin as the most abundant one.

Scientific research has consistently highlighted the significant impact of regularly consuming these molecules on mitigating various age-related diseases, such as cardiovascular diseases, cancer, type 2 diabetes, and obesity [39,40]. These bioactive compounds play a crucial role in promoting overall health and disease prevention, making them valuable components in dietary interventions and preventive strategies.

Currently, the exploration of innovative agricultural food sources has grown rapidly due to the attractive potential for recovering high-value compounds [41]. Various in vitro tests have demonstrated the high potential of different food materials including fennel waste, olive mill wastewater, olive leaves, pea hulls, winemaking waste, broad beans, spent coffee grounds, and more, as promising and sustainable sources of bioactive molecules [42,43,44]. This has led to a great interest in their application in cosmeceutical and nutraceutical contexts [45]. Scientific investigation of these sources opens new avenues for developing environmentally conscious products, driving progress in both the cosmeceuticals and nutraceuticals sectors.

Although previous studies have investigated polyphenolic compounds in zucchini flowers, there has been no prior investigation of a straightforward water-based method of extracting active molecules suitable for nutraceutical products. However, Mohamed et al. [46] investigated the polyphenolic profile of methanolic extracts from zucchini flowers by using an HPLC system with a photodiode-array detector (DAD). The results showed that isorhamnetin, quercetin, and myricetin were the main phenolic components found in this extract. Morittu et al. [47] investigated the polyphenolic fraction of zucchini flower extract using the HPLC-DAD technique. The findings highlighted that rutin, syringic acid, catechin, epicatechin, hesperidin, and quercetin-3-Oglucoside were the predominant phenolic compounds found in the tested extract. In contrast with the literature, the TPC data found in the assayed extracts of zucchini flowers were lower than those reported by Loubet-González [48], who reported a TPC value of 876 mg GAE/100 g DW for methanol extracts of zucchini flowers obtained with Soxhlet. These differences may be related to the different extraction procedures and/or the different solvents used. Moreover, our findings revealed a twofold increase in TPC value compared to the data previously reported by Aquino-Bolaños et al. [49] in ethanol extracts of zucchini flowers.

On the other hand, although scientific research has confirmed the health benefits of polyphenols, it is crucial to recognize their vulnerability to GiD [50]. These plant-based secondary metabolites can undergo significant changes during digestion, affecting their bioaccessibility [20]. Considering the susceptibility of polyphenols to the complexities of digestive conditions, using capsules designed to deliver bioactive compounds to specific target tissues emerges as a promising and strategic approach to advancing dietary supplements and nutraceutical products [51]. Hence, the primary aim of this study was to evaluate how the antioxidant activity and bioaccessibility of polyphenols change during the simulated GiD of two distinct nutraceutical formulations, AcR and NAcR capsules, containing water-based extracts of female zucchini flowers.

Given the limitations of the INFOGEST protocol in mimicking the activity of intestinal microbiota, we used Pronase E and Viscozyme L as alternative enzymes to replicate enzymatic activity in the human large intestine. However, fecal inoculum-based protocols remain the most precise method to replicate microbiota activity during colon digestion [52]; recent research has increasingly supported the practicality and validity of Pronase E and Viscozyme L as an alternative approach to simulating intestinal microbiota activity [53,54].

We measured the TPC value following each GiD phase using the Folin–Ciocâlteu assay to evaluate the bioaccessibility of polyphenols. The results revealed strong correlations between data obtained from ABTS and DPPH assays and TPC values measured during in vitro GiD. These findings confirm the reliability of the conducted assays in providing information about the antioxidant compounds released by the nutraceutical formulations during the simulated GiD process. Our investigation highlighted that in AcR capsules, the bioavailability of polyphenols from zucchini flower extract was 0 mg GAE/g in both the oral and gastric phases, indicating the effective preservation of polyphenols by the capsules during these phases. In contrast, NAcR capsules only showed 0 mg GAE/g of TPC during the oral phase, releasing active compounds during the gastric phase. Moreover, both AcR and NAcR capsules showed higher TPC and antioxidant activity in the duodenal and colonic stages compared to non-encapsulated extracts subjected to GiD (Tables S2 and S3), suggesting the ability of capsules to protect active compounds effectively during the simulated GiD process.

Furthermore, a noteworthy difference emerged when comparing water-based zucchini flower extracts encapsulated in AcR capsules to NAcR samples. AcR capsules exhibited significantly higher antioxidant activity and TPC values in both the duodenal and colonic stages. These results align with prior studies that observed similar high polyphenol bioaccessibility in the colonic stage for water-based extracts enclosed in AcR capsules, such as fennel waste and red cabbage extracts [12,55]. Amrani-Allalou et al. [56] also reported that plant extracts encapsulated in AcR capsules displayed notably higher TPC values following GiD processing compared to the same extracts examined without protective capsules.

These findings demonstrate the remarkable capability of AcR capsules to protect the polyphenol fraction from gastric conditions, preserving the chemical characteristics of bioactive molecules. Consequently, AcR capsules emerge as a promising and effective strategy for facilitating the delivery of active molecules to target tissues, maximizing their nutraceutical potential.

## 5. Conclusions

This research delved deeply into the analysis of polyphenolic components, encompassing phenolic acids and flavonoids, found within water-based extracts derived from female zucchini flowers. Utilizing UHPLC Q-Orbitrap high-resolution mass spectrometry, our investigation unveiled that the most prominent polyphenolic compounds in these extracts were rutin and isorhamnetin-3-rutinoside. Furthermore, our findings indicated that when subjected to GiD, AcR capsules demonstrated the ability to shield these bioactive compounds from adverse gastric conditions. Consequently, this preservation resulted in significantly higher TPC values and enhanced antioxidant activity in both the duodenal and colonic phases compared to NAcR capsules.

These insights emphasize the potential of female zucchini flower aqueous extracts as an innovative source of dietary polyphenols. Additionally, encapsulating the extract in AcR capsules emerges as a promising strategy for effectively transporting antioxidants to targeted tissues. Nonetheless, it is imperative to undertake further investigations aimed at identifying the diverse metabolites generated throughout the GiD process. Finally, further research is required to clarify the several biotransformations of polyphenolic compounds that occur during the GiD process and to broaden our knowledge of the bioactivity of the extracts under study for potential use as components in nutraceutical formulations.

## Figures and Tables

**Table 1:** UHPLC-HRMS parameters.

Analytes	RT (min)	Chemical Formula	Adduct Ion	Theoretical Mass (*m/z*)	Measured Mass (*m/z*)	Accuracy (Δ ppm)	LOD (mg/kg)	LOQ (mg/kg)
Quinic acid	0.47	C_7_H_12_O_6_	[M − H]^−^	191.05531	191.05611	4.18727	0.026	0.078
Protocatechuic acid	2.31	C_7_H_6_O_4_	[M − H]^−^	153.01930	153.01857	−4.77064	0.013	0.039
Chlorogenic Acid	3.00	C_16_H_18_O_9_	[M − H]^−^	353.08780	353.08798	0.50979	0.013	0.039
Epicatechin	3.17	C_15_H_14_O_7_	[M − H]^−^	289.07176	289.07202	0.89943	0.013	0.039
Caffeic acid	3.23	C_9_H_8_O_4_	[M − H]^−^	179.03498	179.03455	−2.40177	0.013	0.039
Catechin	3.34	C_15_H_14_O_6_	[M − H]^−^	289.07175	289.07205	1.03780	0.026	0.078
*p*-Coumaric acid	3.46	C_9_H_8_O_3_	[M − H]^−^	163.04001	163.03937	−3.92542	0.013	0.039
Vitexin	3.48	C_21_H_20_O_10_	[M − H]^−^	431.09837	431.09711	−2.92277	0.013	0.039
Apigenin-7-O-glucoside	3.49	C_15_H_10_O_5_	[M − H]^−^	269.04555	269.04526	−1.07788	0.026	0.078
Ferulic acid	3.55	C_10_H_10_O_4_	[M − H]^−^	193.05063	193.05016	−2.43459	0.026	0.078
Naringin	3.56	C_27_H_32_O_14_	[M − H]^−^	579.17193	579.17212	0.32805	0.013	0.039
Quercetin-3-galactoside	3.58	C_21_H_20_O_12_	[M − H]^−^	463.08820	463.08817	−0.06478	0.039	0.117
Rutin	3.59	C_27_H_30_O_16_	[M − H]^−^	609.14611	609.14673	1.01782	0.013	0.039
Diosmin	3.64	C_28_H_31_O_15_	[M − H]^−^	607.16684	607.16534	−2.47049	0.013	0.039
Kaempferol-3-glucoside	3.68	C_21_H_20_O_11_	[M − H]^−^	447.09195	447.09329	2.99715	0.013	0.039
Isorhamnetin-3-rutinoside	3.72	C_28_H_32_O_16_	[M − H]^−^	623.16176	623.16174	−0.03209	0.026	0.078
Myricetin	3.73	C_14_H_10_O_8_	[M − H]^−^	317.03029	317.02924	−3.31199	0.013	0.039
Daidzein	3.77	C_15_10_0_O_4_	[M − H]^−^	253.05063	253.05035	−1.10650	0.013	0.039
Quercetin	3.88	C_15_H_10_O_7_	[M − H]^−^	301.03538	301.03508	−0.99656	0.013	0.039
Naringenin	3.91	C_15_H_12_O_5_	[M − H]^−^	271.06120	271.06110	−0.36892	0.013	0.039
Luteolin	3.98	C_15_H_10_O_6_	[M − H]^−^	285.04046	285.04086	1.40331	0.026	0.078
Kaempferol	4.01	C_15_H_10_O_6_	[M − H]^−^	285.04046	285.04086	1.40331	0.013	0.039
Genistein	4.05	C_15_H_10_O_5_	[M − H]^−^	269.04554	269.04562	0.29735	0.013	0.039
Apigenin	4.08	C_15_H_10_O_5_	[M − H]^−^	269.04555	269.04556	0.03717	0.039	0.117

**Table 2:** Polyphenolic compounds in zucchini flower water extract. Results are expressed as mean ± SD (of three repetitions, *n* = 3).

Analytes	Average (mg/kg)	±SD
Rutin	514.62	4.17
Isorhamnetin-3-rutinoside	318.59	2.55
*p*-Coumaric acid	63.69	0.21
Quercetin-3-galactoside	51.25	0.18
Kaempferol-3-glucoside	39.61	0.19
Myricetin	16.37	0.16
Quercetin	14.02	0.06
Kaempferol	6.75	0.09
Apigenin	1.18	0.03
Naringin	<LOQ	

Abbreviations: LOQ: limit of quantification. The other analytes investigated were not detected.

**Table 3:** Bioaccessibility of polyphenols (of three repetitions, *n* = 3) assessed by the Folin–Ciocâlteu method in undigested zucchini flower water extract and during simulated in vitro digestion of encapsulated formulations.

Samples	TPC mg GAE/100 g ± SD	
Not Digested	534.2 ± 18.3	
	NAcR	AcR
Digestion Stage		
Oral stage	n.d	n.d
Gastric stage	64.6 ± 3.2	n.d
Duodenal stage	162.5 ± 7.1	189.2 ± 8.5
Pronase E	212.7 ± 9.6	263.5 ± 16.5
Viscozyme L	132 ± 9.2	163.9 ± 6.3
Total colonic stage	344.7 ± 9.4	477.4 ± 11.6

Abbreviations: TPC: total phenolic compounds; NAcR: non-acid-resistant; AcR: acid-resistant; n.d.: not detected.

**Table 4:** Antioxidant capacity measured by DPPH and ABTS tests. Results were expressed as mmol Trolox equivalents per kg (of three repetitions, *n* = 3).

Not Digested	DPPH mmol Trolox/kg ± SD		ABTS mmol Trolox/kg ± SD	
	12.6 ± 0.3		15.9 ± 0.3	
	AcR	NAcR	AcR	NAcR
Digestion stage				
Oral stage	n.d	n.d	n.d	n.d
Gastric stage	n.d	1.4 ± 0.2	n.d	1.9 ± 0.2
Duodenal stage	2.6 ± 0.2	1.8 ± 0.2	3.7 ± 0.3	2.8 ± 0.1
Pronase E stage	3.6 ± 0.2	2.3 ± 0.1	4.2 ± 0.1	3.6 ± 0.2
Viscozyme L stage	1.5 ± 0.2	1.5 ± 0.1	2.1 ± 0.2	1.8 ± 0.1
Total colonic stage	5.1 ± 0.2	3.8 ± 0.1	6.3 ± 0.2	5.4 ± 0.2

Abbreviations: NAcR: non-acid-resistant; AcR: acid-resistant; n.d.: not detected.

## Data Availability

Data is contained within the article and supplementary material.

## Supplementary Materials

The following supporting information can be downloaded at: https://www.mdpi.com/article/10.3390/antiox12122108/s1, Table S1: Total phenolic content in CT-AcR and CT-NAcR formulations; Table S2: Bioaccessibility of polyphenolic compounds in non-encapsulated zucchini flower extracts subjected to GiD process; Table S3: Antioxidant capacity measured by DPPH and ABTS tests in non-encapsulated zucchini flower extracts subjected to GiD process. Results are expressed as mmol Trolox equivalents per kg; Table S4: Correlation between TPC and data obtained by DPPH and ABTS tests. Correlation coefficients were evaluated by using Pearson’s method; Table S5: Antioxidant activity evaluated by ABTS and DPPH tests in CT-AcR and CT-NAcR formulations.
