# Supplementary material for: Exploring the Chemical Composition of Female Zucchini Flowers for Their Possible Use as Nutraceutical Ingredient
Source: Antioxidants (Basel). 2023 Dec 13;12(12):2108. doi: 10.3390/antiox12122108 (PMC10740746; doi:10.3390/antiox12122108)
Supplement: Supplementary file 1 [file antioxidants-12-02108-s001.zip › antioxidants-2760589-supplementary.pdf]

antioxidants

MDPI

Supplementary materials:

Table S1. Total phenolic content in the CT-AcR and CT-NAcR formulations.

**Samples**	**TPC mg GAE/100g±SD**	
Cellulose	0.2±0.00	
	**CT-NAR**	**CT- AR**
Digestion Stage		
Oral stage	n.d	n.d
Gastric stage	n.d	n.d
Duodenal stage	n.d	n.d
Pronase E	n.d	n.d
Viscozyme L	n.d	n.d
Total colonic stage	n.d	n.d

Table S2. Bioaccessibility of polyphenolic compounds in not-encapsulated zucchini flower extracts subjected to GiD process.

**Sample**	**TPC mg GAE/100g±SD**
Not digested	534.2±18.3
Digestion Stage	
Oral stage	134.2±0.2
Gastric stage	56.4±0.4
Duodenal stage	154.5±0.5
Pronase E	136.9±0.4
Viscozyme L	113.2±0.6
Total colonic stage	250.1±0.5

Abbreviations: mg GAE/100g: milligrams of gallic acid equivalent per 100 grams of dry extract.

antioxidants

MDPI

Table S3. Antioxidant capacity measured by DPPH, and ABTS tests in not-encapsulated zucchini flower extracts subjected to GiD process. Results were expressed as mmol trolox equivalents per kg.

	DPPH mmol trolox/kg±SD	ABTS mmol trolox/kg±SD
**Not digested**	12.6±0.3	15.9±0.3
Digestion stage		
Oral stage	1.4±0.2	2.1±0.1
Gastric stage	1.3±0.2	1.8±0.3
Duodenal stage	1.2± 0.1	1.6± 0.2
Pronase E stage	1.1±0.2	1.4±0.2
Viscozyme L stage	1.2±0.2	1.3±0.2
Total colonic stage	2.3±0.2	2.7±0.2

Abbreviations: mg GAE/100g: milligrams of gallic acid equivalent per 100 gram of dry extract.

Table S4. Correlation between TPC and data obtained by the DPPH, and ABTS tests. The correlation coefficients were evaluated by using Pearson's method.

**Test**	**Gastric Stage**	**Duodenal Stage**	**Pronase Stage**	**Viscozyme Stage**
	R^2	R^2	R^2	R^2
DPPH	0.98	0.98	0.98	0.97
ABTS	0.97	0.98	0.96	0.98

Table S5. Antioxidant activity evaluated by ABTS and DPPH tests in CT-AcR and CT-NAcR formulations.

	**DPPH mmol trolox/kg±SD**		**ABTS mmol trolox/kg±SD**	
**Cellulose not-digested**	0.3 ± 0.0		0.2 ± 0.0	
	**CT- AcR**	**CT- NAcR**	**CT- AcR**	**CT- NAcR**
Digestion stage				
Oral stage		n.d.	n.d.	n.d.
Gastric stage	n.d.	n.d.	n.d.	n.d.
Duodenal stage	0.1 ± 0.0	n.d.	0.1 ± 0.0	n.d.
Pronase E stage	n.d.	0.1 ± 0.0	n.d.	n.d.
Viscozyme L stage	n.d.	n.d.	n.d.	n.d.
Total colonic stage	n.d.	0.1 ± 0.0	n.d.	n.d.
